# Consumption of Genetically Modified Food Products and Its Determinants (Case Study: Edible Oil in Mashhad)

**DOI:** 10.3390/foods12152933

**Published:** 2023-08-02

**Authors:** Reihaneh Zhaleh, Hosein Mohammadi, Flavio Boccia, Ali Firoozzare, Daniela Covino

**Affiliations:** 1 Department of Agricultural Economics, College of Agriculture, Ferdowsi University of Mashhad, Mashhad 91779-48978, Iran; reyhane.zhale@gmail.com (R.Z.); firooz@um.ac.ir (A.F.); 2 Department of Economic and Legal Studies, Parthenope University of Naples, 80132 Naples, Italy; daniela.covino@uniparthenope.it

**Keywords:** genetically modified, food products, edible oil, marketing mix, Tobit model, consumption

## Abstract

In recent decades, the global increase in the demand for food and the increasing growth of the world population has caused an inevitable transition from traditional to advanced agriculture and the use of new technologies in the production of food and agricultural products. One of the new achievements of biotechnology is the production and use of genetically modified plants. The benefits of genetically modified crops can be seen well beyond the farm as well, from helping to conserve natural resources to fighting climate change. Identifying the factors that influence people’s acceptance of genetically modified (GM) foods can inform industries and policymakers regarding their innovation trajectories, as well as policy development and implementation. Therefore, the current research evaluates the effect of the marketing mix and other effective factors on the consumption of genetically modified (GM) edible oil in Mashhad, Iran. The required information was collected by completing 390 questionnaires and using the available sampling method in 2022. Factors affecting the probability of consumers making a decision to consume GM edible oil and the consumption amounts of this oil were analyzed through Heckman’s two-stage Tobit model using the STATA 16 software package. The results showed that factors affecting the intention of consuming GM edible oils are different from factors affecting the amount of consumption of GM edible oils. Moreover, selected marketing mixes have a significant effect on the amount of consumption of GM edible oils, and therefore, policy-makers can influence the consumption of GM edible oils by using marketing tools. The effect of household monthly income on the consumption of GM edible oil is also negative and significant, which shows that households with higher incomes have less consumption of GM edible oils. Based on the results, trust in the government has a positive and significant effect on the consumption of GM edible oil, so when consumers have trust in their government about GM food products, the consumption of GM edible oil will increase. Therefore, it is suggested that the country’s food security authorities gain the trust of consumers by clarifying the production process of GM products and holding scientific debates between the proponents and opponents of the production and consumption of GM food products in order to express the advantages and disadvantages of these products to inform consumers and help them choose between products.

## 1. Introduction

Today, food security is one of the most important challenges in facing climate change and population growth [1] and while the world’s demand for food, grains and animal protein is increasing, the conventional agriculture cannot keep up with what is needed [2]. Therefore, the increase in the world population along with increasing growth in food demands in recent decades has caused an inevitable transition from traditional to advanced agriculture and has necessitated the use of new technologies in the field of food and agricultural sciences. One of the new achievements of biotechnology is the production and use of genetically modified (GM) plants [3].

The World Health Organization (WHO) defines genetically modified organisms (GMOs) as organisms (i.e., plants, animals or microorganisms) in which the genetic material (DNA) has been altered in a way that does not occur naturally through mating. The foods which are derived from GM organisms are often referred to as GM foods [4]. These biotechnological innovations have great potential to achieve sustainable food development and food security, and they are expected to play an important role in feeding the growing world population [5] and meet forthcoming global needs in the near future without putting additional pressure on the environment [2].

In short, the advantages of GM food products can be mentioned as providing food security, protecting biodiversity, reducing the consumption of environmental pollutants in agriculture, eliminating poverty and hunger and, most importantly, contributing to sustainable economic growth [6]. With the emergence of desirable traits in crops, biotechnology has paved the way for the participation of genetically modified (GM) crops in sustainable food production systems [7]. According to the prediction, by 2050, GM crops will be cheaper than other agricultural crops, so that these crops will be easily available due to their special capabilities in increasing yield and sustainability [8].

In the last 25 years, GM crop production has experienced an over 100-fold increase [9] and currently farmers cultivate approximately 190 million hectares of biotech crops [10]. Soybean (~50%), maize (~30%), cotton (~13%) and canola (~5%) are the four primary cultivated crops [4]. United States with the cultivation of 75 million hectares, had the highest level of GM crops in the world in 2019, and then Brazil is in second with 52.8 million hectares [11]. Currently, there are at least 32 approved GM crops consisting of 24 crops intended for foods or consumable products (e.g., vegetable oils), while the others are non-food crops, such as alfalfa, cotton, and ornamental flowers [12].

Genetic modification of crops has substantially focused on improving traits for desirable outcomes, although its widespread adoption faces several challenges due to concerns about human health, the environment and moral issues [7]. Furthermore, GM products are still new products in the market, and consumers may be more hesitant to buy them.

Trying to understand what goes on in a consumer’s head and exactly what makes them buy is a goal of every business. The only way to do this is by closely studying the buying patterns and by building theories and models. Consumer behavior theory is the study of how people make decisions when they purchase, helping businesses by predicting how and when a consumer will make a purchase. It helps to identify what influences these decisions, as well as highlight strategies to proactively manipulate behavior [13]. Customer behavior is shaped by a few key factors of psychological (e.g., a person’s attitude), personal (e.g., age, gender), and social (e.g., education). Consumer behavior theory allows businesses to understand more about their target audience and so be able to craft products, services and company culture to influence buying [14].

Several factors affect the choice and consumption of a product [15], and consumer attitudes toward technology and GM foods vary across cultures and geographic characteristics around the world [16]. Previous studies showed that subjective norms relating to the acceptance of GM products, attitudes of consumers and perceived risks and benefits are the main determining factors for the consumption of these types of products [17]. For instance, some consumers are concerned about harmful aspects of GM foods [18], while for others, health and environmental concerns may be effective in the formation of attitudes [19].

A number of studies have shown that some Asian (i.e., Japan and Taiwan) and European consumers have difficulties embracing GM products. This is due to consumers’ concern about the unknown effects of transgenic foods on human health and religious and moral issues [20]. Moreover, the attitude of Lithuanian and Georgian consumers towards GM products is negative [21,22].

According to attitudes, women with higher education levels have a more negative attitude toward GM products than others [23]. In addition, the women reported that they receive information about genetically modified food products through television and radio [23]. Norwegian women are more skeptical of GM crops than men, and older people need more discounts to buy GM products, which means that older consumers are less receptive to GM products than younger people [24], and public trust in government and belief in science is increased by positive media influence and increases in public support [25]. Moreover, in order to improve the effectiveness of food policies, policymakers should invest in advertising labels and target the elderly and people with a lower education levels [26].

Health concerns reduce trust in GM foods, but trust exerts a positive influence on consumers’ intentions toward these products [27]. Consumer trust in biotechnology research institutes, government departments and relevant experts in the field of GM products can more easily encourage customers’ intention to purchase GM products such as soybean oil [28]. The acceptance of GM foods by consumers who do not trust the government is low, and those who are more aware of these products are more likely to accept GM-labeled foods [29]. Chinese consumers have little trust in the government, biotech scientists, and the press, and they believe that the information they receive is limited and does not clearly show the advantages and disadvantages of GM food [30]. Expert organizations could highlight the scientific consensus on the safety of GM foods and reduce false consensus among the public, which leads to reduced misconceptions of GM foods and strengthening consumption behaviors [31].

The role played by education in improving people’s understanding of the issues associated with GM foods provides insights to assist marketers in developing differentiated strategies. Marketers would be able to help consumers dampen the effect of fear and allow them to develop more informed opinions [32]. The acceptance of novel technology is shown to correspond closely to the degree of consumers’ scientific knowledge, highlighting the importance of revealing relevant information regarding the technology [33]. People with more knowledge about GM products consume more of these products [34], and the age, gender and marital status of consumers with higher education are factors that affect their knowledge and attitude about GM foods [18]. Information-seeking health-conscious consumers tend to be less likely to purchase GM foods, while more money-conscious consumers are more likely to purchase GM products [35]. Due to the lack of consumer knowledge about GM food consumption, it seems that these foods have a negative effect on consumer perception [36]. Consumer loyalty towards GM food is affected by the interaction between the awareness of benefits and risks, situational and social influences, and attitude and repurchase intention [37].

Based on the results of [38], producers of edible oil should pay more attention to factors such as price advantages, advertising, product accessibility and, of course, quality and branding. Price and advertising have the strongest effect on consumer acceptance and purchase [39,40,41] of food products. In Alabama, if the price difference between GM and non-GM tomatoes increases, the probability of consumers buying GM tomatoes increases [42].

Product factors such as product quality, brand, product taste and health and environmental safety are also important for consumers when choosing food products [43]. Location factors are less important for consumers compared to the product, but the results of [33,40] show that the country of origin is an effective factor on consumer food purchasing decisions. The authors of [44] show that trust in GM foods, and concerns about the health and environmental impacts of GM foods, predicted the intention to consume such products. Another important aspect of GM food production is the significant reduction in the release of greenhouse gas emissions from GM cropping areas, which has been shown to lead to a decrease in carbon emissions from cropping agriculture [45].

Based on the results of [46], the brand name of edible oils is more important than other factors, because consumers think that familiar product names have better quality than unfamiliar products. Also, the GM information included on the edible oil label had a negative effect on the purchase intention of consumers and caused them to reject GM soybean oil. The research results of [47] showed that there is no significant difference regarding the acceptance of GM milk according to the gender of people, but the most sensitive consumers to GM food products are people over 35 years old and without children.

GM crops are not cultivated in Iran, and despite the successful production of some GM plants in field experiments by researchers, no GM domestic products have been approved for sale. On the other hand, Iran is an importer of oil, fodder, and corn, and it is highly dependent on the import of the world’s main GM products (soybean, cotton, corn, and canola). In 2018, Iran’s Ministry of Health, Medicine and Medical Education declared only three oilseed products, namely, rapeseed, soybean and corn, as allowed GM products in the country and stated that these products must have labels. Also, more than 6 million tons of animal inputs are imported into the country annually, including GM corn [1]. According to the statistics of Iranian customs, the import of crude edible oils in 2022 has increased compared to previous years, and sunflower, soybean and palm oils have been imported to the country in higher quantities than other oils in 2022.

Due to the increase in the production and consumption of GM foods in recent years and the importance of investigating the impact of factors affecting the consumption of these products, several studies have been conducted in this regard in Iran and in the world. Most of the studies conducted in the field of factors influencing the consumption of GM foods by consumers have used variables such as age, gender, number of household members, education level, household income, etc. The innovation of this research is that, in addition to the mentioned variables, we also investigate the effect of marketing mix on the probability of buying and the consumption of GM foods (GM edible oil). The marketing mix consists of the famous four Ps of marketing: product, place (to distribute or deliver the product to the consumer), price and promotion [48]. Marketing mix plays an important role in influencing consumers to buy products or services offered by the market, which ultimately indicates the degree of marketing success [49]. In addition, another contribution of this study is the use of Heckman’s two-stage Tobit model to distinguish between factors affecting the intention to consume GM food products (edible oil) and the amount of consumption of GM food products, which is less mentioned in related studies. Therefore, the hypothesis of the research are as follows:

**Hypothesis 1 (H1).**  *Selected marketing mix have a significant effect on the amount of consumption of GM food products.*

**Hypothesis 2 (H2).**  *Factors affecting on the intention of consuming GM food products are different to factors affecting on the amount of consumption of GM food products.*

This study aimed to investigate the factors affecting the consumption of GM food products (edible oil) between consumers in Mashhad and to identify the key factors influencing the amount of GM edible oil consumption between households. To achieve this goal, we first designed a questionnaire containing information about the individual characteristics of consumers and the important factors affecting consumption of GM food products, and then we conducted a survey of 390 consumers in Mashhad, Iran, in 2022. To this end, in the next section, we present the details of the methodology used, the study design, data collection and data analysis, and the methods of estimation. The next sections present the results, discussions, main conclusions and policy implications. Our findings show that policy-makers can use marketing mix for changing consumers’ behavior according to the consumption of GM edible oils or other GM food products.

## 2. Methodology

The statistical population of this research includes all heads of households in Mashhad city in Iran. The Mashhad population was approximately about 3.3 million people in 2020. To check the statistical population of the research, the 13 districts of Mashhad municipality are classified into 5 levels of development based on sustainable urban development indicators. Then, according to the sample size and Morgan’s table, 390 questionnaires were completed in 2022 with the available sampling method and with appropriate allocation of the population of each development level.

The data collection tool in this research is a questionnaire whose questions and variables are designed based on consumer behavior theory, similar studies, and the literature. The questionnaire was designed with both open and closed questions, with limited multiple-choice options, and depending on the type of variables, binary or ordered scales are considered for variables and answers.

According to the pre-tests, the sample is a suitable representative for the population, and the response rate was 23%. The validity of the questionnaire was checked by experts and professors, and the necessary corrections were applied. Cronbach’s alpha was also used to check the reliability of the questionnaire [50,51].

In this study, we used the two-stage Heckman regression approach to investigate factors affecting consumption of GM edible oil. The reason for using this model is that logit or probit models do not have the ability to distinguish between factors that influence decisions to consume GM food products, as well as factors that influence the level of consumption of GM food products. The Tobit model utilizes observation of both groups of potential consumers of GM food products and actual consumers of GM food products to resolve Type I error (non-random sampling). However, it does include the risk of Type II error (lack of differentiation between the factors affecting the decision to consume GM food products and the factors affecting the amount of consumption of GM food products). Heckman suggested a two-step method for resolving the second problem. Heckman’s two-step method is based on the assumption that a set of variables can affect the decision to consume GM food products, and another set of variables can affect the volume of consumption of GM food products after making the initial decision. Hence, the two groups of variables are not necessarily similar [52].

The structure of the Tobit model is expressed as follows:

(1) $$\begin{array}{l} Y_{i}=\beta^{\prime }X_{i}+U_{i}{}_{}{}_{}{}_{}{}_{}\;\;\;Y_{i}^{*}>0\\ Y_{i}=0{}_{}{}_{}{}_{}{}_{}{}_{}{}_{}{}_{}{}_{}\;\;\;\;\;\;Y_{i}^{*}\leq 0\\ i=1,\ldots ,n \end{array}$$

where $Y_{i}^{*}$ is the latent variable, $Y_{i}$ is the observed variable, $\beta^{\prime }$ is the vector of model parameters, $X_{i}$ is the vector of independent variables, $U_{i}$ is the disturbance term, and n is the total number of observations [53]. For consumers who consume GM edible oil, $Y_{i}^{*}$ is consumption level, while for consumers who do not consume GM edible oil, $Y_{i}^{*}$ is zero. Thus, the cutting threshold was zero [53].

Accordingly, the first step is to estimate a model that shows the probability of consuming GM edible oil, and for this part, the probit regression model was used as shown below:

(2) $$\begin{array}{l} Z_{i}=\beta^{\prime }X_{i}+v_{i}{}_{}{}_{}{}_{}{}_{}\;\;\;i=1,2,\ldots n\\ Z_{i}=1{}_{}{}_{}{}_{}{}_{}^{}{}_{}{}_{}{}_{}\;\;\;\;\;\;if\;{}_{}Y_{i}^{*}>0\\ Z_{i}=0{}_{}{}_{}{}_{}{}_{}{}_{}{}_{}{}_{}\;\;\;\;\;\;if\;{}_{}Y_{i}^{*}<0 \end{array}$$

$Z_{i}$ is the dependent variable of the first step. If a household consumes GM edible oil, its value is 1; otherwise, its value is zero. The first step estimates factors affecting a household’s decision to consume GM edible oil. The inverse Mills ratio (IMR), $\lambda =\frac{\phi (\beta^{\prime }X_{i})}{\varphi (\beta^{\prime }X_{i})}$ is the ratio of the standard normal density function to the standard normal cumulative distribution function [54].

In the second step, the relationship between the independent variables and the amount of GM edible oil consumption is estimated using observations of $Y_{i}$ on $X_{i}$ and IMR, which are obtained from the first step of probit analysis:

(3) $$Y_{i}=\beta^{\prime }X_{i}+\sigma IMR_{i}+e_{i}$$

The second estimation shows how the explanatory variables affect consumption levels for GM edible oil. The IMR coefficient measures errors resulting from sampling, and if they are significantly different to zero, it indicates bias in the sampling [55]. The presence of the inverse Mills ratio variable in the above linear regression model removes the variance heteroscedasticity of the initial model and permits the use of the ordinary least squares estimator [56].

## 3. Results 

Descriptive statistics of explanatory variables in the study are given in Table 1.

Next, the respondents were asked whether they consume GM edible oil such as corn, canola, soybean, etc., or not. About 32.3 percent of the respondents stated that they do not use GM edible oils at all, and 67.7% of the respondents consume a small or large amount of these types of edible oils (Table 2).

Then, the people who consume GM edible oil were asked how much GM edible oil they consume per month (in liters). The results are shown in Table 3.

The results of the estimation of the first stage of Heckman’s two-stage Tobit model are shown in Table 4.

Based on the results of Table 5, the source of information, monthly household expenditure, discounts on GM edible oil, advertisements, packaging and, oil-producing country have a positive and significant effect on the probability of consumers making a decision to consume GM edible oil. Also, the variables of consumer’s knowledge regarding GM products, free flow of information in the society, quality of other edible oils, the place of supply and price levels have a negative and significant effect on the probability of consumers making a decision to consume GM edible oil.

Several types of coefficient of determination ($R^{2}$) as a measure of goodness of fit have been proposed for limited dependent variables [54]. These types of $R^{2}$ do not have an interpretation like $R^{2}$ in the linear regression. The results of goodness of fit measures of the first stage Probit model are reported in table.

The results of the estimation of the second stage of two-stage Heckman’s Tobit model, that is, the linear regression model and the introduction of variables affecting the consumption level of GM edible oil, is reported in Table 6.

As the results of the linear regression model Table 6 show, the inverse Mills ratio variable was added to the explanatory variables in the regression model.

The results in Table 6 show that the effect of number of family members, monthly food expenditure, discounts, advertisement and trust in the government on the level of GM edible oil consumption is positive and significant, while the effect of education, monthly income, information, and brand of other edible oils (not GM) on the level of consumption of GM edible oils is negative and significant. According to the results, the coefficient of determination in this model is 0.286; thus, it can be said that about 28.6% of the GM edible oil consumption level is explained by the variables used in the model.

To check the residual autocorrelation, the Durbin–Watson statistic for the linear regression model was calculated and is equal to 1.988, which indicates that there is no autocorrelation between residuals of the estimated model. In order to investigate the multi-collinearity between variables, the variance inflation factor (VIF) test can be used [54]. Variance inflation factor (VIF) measures how much the behavior (variance) of an independent variable is influenced, or inflated, by its interaction/correlation with the other independent variables. If the VIF value is between 1 and 5, it indicates a weak correlation between explanatory variables [55]. Given that the VIF value of the model is equal to 1.29, there is therefore no multi-collinearity between the explanatory variables. 

Finally, the White test was used to check the variance of the residuals in the estimated model. The result of White test indicate that the variance of residuals is homogenous, and therefore, using the OLS method in the second stage of model estimation was correct [57].

## 4. Discussion

To interpret the coefficients of the first stage of the Tobit–Heckman model, the marginal effects should be used. According to the results of the first stage of the Heckman model, one of the effective marketing tools for the probability of consuming GM edible oil is products with quality and packaging dimensions. The marginal effect of quality in the Probit model is negative and significant, which means that with an increase in the quality of other edible oils in society, the probability of consumers making a decision to consume GM edible oil will decrease by 9%. Also, the effect of the packaging of GM edible oil on the probability of consumers deciding to consume these types of oils is positive, which means that by improving the packaging, the probability of consumers deciding to consume GM edible oil will increase by 21.7%.

Another effective marketing mix on the probability of consumers deciding to consume GM edible oil is the price and price discounts. The marginal effect of GM edible oil price is −0.127, which indicates the negative effect of price level on the consumption of GM edible oils, and it is consistent with the consumer behavior theory. Moreover, the marginal effect of price discount is 0.076 and significant, which shows the positive effect of this variable on making a decision to consume GM edible oil.

Promotion is also an effective factor in the possibility of consumers deciding to consume GM edible oils. The advertisement of GM edible oils has a positive and significant effect on the probability of consumers making a decision to consume GM oil, and with advertisement, the probability of consumers making a decision to consume GM oil will increase by 35.6%.

The last marketing mix that is effective on the probability of consumers deciding to consume GM edible oil is distribution place and oil-producing country. The estimated marginal effect for place of supply is −0.136 and significant, which means that this variable has a negative effect on the possibility of consumers deciding to consume GM edible oils. Moreover, GM oil-producing country is an important factor in consumers deciding to consume GM edible oil.

Based on the results of the first stage of the model, we prove that marketing mix has a significant effect on consumers’ making a decision regarding the consumption of GM edible oils. Therefore, our first hypothesis is confirmed. According to [58], the combinations of price, product, advertisement, and location are effective on the purchasing behavior of the respondents.

According to the results of the second stage of model estimation, the inverse Mills ratio variable is statistically significant at the 10% level, and it shows that the factors affecting the probability of consumers making a decision to consume GM edible oil are not the same as the factors affecting the level of consumption of GM edible oil, which validates our using Heckman’s two-step method in this research. Therefore, this result indicates that our second hypothesis is confirmed.

The results of estimation in the second stage of Heckman’s model show that if the number of family members increases by one person, the amount of GM edible oils increases by 0.09 percent, while if the level of education of the head of households increases, the amount of GM edible oils decreases by 0.10 percent. The effect of household monthly income on the consumption of GM edible oil is negative and significant, which shows that households with higher incomes have less consumption of GM edible oils. Therefore, these types of oils are inferior goods, and by increasing incomes, their consumption would decrease.

Also, the effect of oil brands on the consumption level of GM edible oil is negative and significant, which indicates that if producers of non-GM edible oil can establish powerful brands, the amount of consumption of GM edible oil in the society will decrease.

As in the first stage, the effect of price discount and advertisement on the level of consumption of GM edible oil is positive and significant, while the effect of GM edible oil price level is negative and significant.

Based on the results, trust in the government has a positive and significant effect on the consumption of GM edible oil, so when consumers have trust in their government about GM food products, the consumption of GM edible oil will increase by 14.5%. According to the research results of [59], if consumers do not have the necessary trust in GM rice producers in the country, it will be difficult to accept this product in society. Therefore, it is suggested that the country’s food security authorities gain the trust of consumers by clarifying the production process of GM products.

## 5. Conclusions and Suggestions

In this research, the factors affecting the consumption of GM edible oil were considered, with an emphasis on marketing mix using the two-stage Heckman model. According to the results of the first stage of the Tobit model, people’s knowledge about GM products has a negative effect on the possibility of consumers making a decision to consume GM edible oil. Also, advertisements have a positive and significant effect on the possibility of consumers making a decision to consume GM edible oil.

According to the results, one of the effective marketing tools for the probability of consuming GM edible oil is products with quality and packaging dimensions. Because product quality and packaging have a significant impact on the probability of consuming GM edible oil, it is suggested that more research and innovation be devoted to improving the quality and packaging of GM products.

Another effective marketing mix on the probability of consumers deciding to consume GM edible oil is price and price discounts, which is consistent with consumer behavior theory. Therefore, producers of GM edible oils can use price discount policy and pricing tools to increase the consumption of GM edible oil in society.

Promotion is also an effective factor in the possibility of consumers deciding to consume GM edible oils. Therefore, producers of GM edible oils can use targeted advertisement to increase the consumption of GM edible oil in society.

The last marketing mix that is effective on the probability of consumers deciding to consume GM edible oil is distribution place and oil-producing country. Therefore, wide distribution of GM products and importing GM products from reputable and well-known countries can increase the consumption of this products.

According to the results of the second stage of model estimation, factors affecting the probability of consumers making a decision to consume GM edible oil are not the same as the factors affecting the level of consumption of GM edible oil.

The results of estimation in the second stage of Heckman’s model show that the effect of the number of family members, monthly food expenditure, discounts, advertisement and trust in the government on the level of GM edible oil consumption is positive and significant, while the effect of education, monthly income, information, and brand of other edible oils (not GM) on the level of consumption of GM edible oils is negative and significant.

The effect of household monthly income on the consumption of GM edible oil is negative and significant, which shows that GM edible oils are inferior goods, and by increasing incomes, their consumption would decrease. Therefore, countries with higher per capita incomes should investigate and substitute more nutrition-rich and healthy edible oils with GM edible oil.

Also, the effect of oil brands on the consumption level of GM edible oil is negative and significant, which indicates that if producers of non-GM edible oil can establish powerful brands, the amount of consumption of GM edible oil in the society will decrease.

Based on the results, trust in the government has a positive and significant effect on the consumption of GM edible oil, so when consumers have trust in their government about GM food products, the consumption of GM edible oil will increase. Therefore, it is suggested that the country’s food security authorities gain the trust of consumers by clarifying the production process of GM products.

If consumers do not have the necessary trust in the producers of GM products in the country, it will be difficult for consumers to accept these products in society. According to the importance of this issue, it is suggested that the government have clear and ongoing communication with experts and researchers in the field of GM products. On the other hand, people are aware of the lack of supervision and implementation of control laws regarding food, so they distrust those in charge of the country’s food security. Therefore, it is necessary for these officials to gain the trust of consumers by clarifying the process of producing GM food products.

## Figures and Tables

**Table 1:** Descriptive statistics for explanatory variables.

Variable	Mean	SD	Min	Max	Description
Gender of household head	0.57	0.49	0	1	Women = 0, and men = 1
Age of household head	44.4	11.9	20	84	Years
Education of household head	2.7	0.88	1	4	Sub-Diploma = 1, Diploma = 2 Bachelor = 3, M.Sc. and above = 4
Monthly income	0.7	0.45	0	1	More than 9 million Tomans or USD 316 per month = 1, otherwise = 0
Monthly expenditure	0.63	0.48	0	1	More than 8 million Tomans or USD 281 per month = 1, otherwise = 0
Number of family members	3.6	1.29	1	8	Count
Area of residence (based on income level)	2.2	1.45	1	5	Very unstable = 1 Unstable = 2 Medium stability = 3 Stable = 4 Very stable = 5
Monthly share of food expenses from monthly expenditure	0.78	0.15	0	1	More than 40% = 1, Otherwise = 0
Familiarity with GM products	0.67	0.29	0	1	Familiar = 1, Otherwise = 0
Trust in the government	0.45	0.21	0	1	Having trust = 1, Otherwise = 0
The effect of place of supply on the consumption	0.69	0.29	0	1	Important = 1, otherwise = 0
The effect of edible oil advertisment on the consumption	0.64	0.22	0	1	Important = 1, otherwise = 0
The effect of edible oil brand on the consumption	0.59	0.33	0	1	Important = 1, otherwise = 0
The effect of discounts on GM edible oil consumption	0.56	0.21	0	1	Important = 1, otherwise = 0
The effect of consumer knowledge on GM edible oil consumption	0.51	0.28	0	1	Important = 1, otherwise = 0
The effect of price of GM edible oil consumption	0.68	0.26	0	1	Important = 1, otherwise = 0
The effect of quality on the consumption of edible oils	0.43	0.29	0	1	Important = 1, otherwise = 0
The effect of edible oil-producing country on the consumption	0.41	0.19	0	1	Important = 1, otherwise = 0
The effect of packaging on the consumption	0.38	0.15	0	1	Important = 1, otherwise = 0
The effect of edible oil quality on the consumption	0.44	0.26	0	1	Important = 1, otherwise = 0
The effect of source of information on the consumption	0.36	0.3	0	1	Important = 1, otherwise = 0
Free flow of information in the society	0.55	0.25	0	1	Important = 1, otherwise = 0

**Table 2:** Dependent variable in the first stage of the Tobit model.

Consumption of GM Edible Oil	Number	Percentage
Consumption of of GM edible oil = 1	264	67.7
Not consumption of GM edible oil = 0	126	32.3

**Table 3:** Dependent variable frequency in the second stage of the Tobit model.

The Amount of GM Edible Oil Consumed per Month	Number	Percentage
Less than 1 L	14	3.6
Between 1 and 2 L	210	53.8
Between 3 and 4 L	32	8.2
More than 4 L	8	2.1
Total	264	67.7

**Table 4:** Estimation results of the first stage of the Tobit model.

Variables	Coefficients	Standard Deviation	T Stat.	Prob.	Marginal Effect
Gender	−0.128	0.148	−0.86	0.389	−0.043
Age	0.009	0.006	1.56	0.119	0.003
Residential area	−0.075	0.050	−1.50	0.134	−0.025
Monthly expenses	0.350 ***	0.151	2.31	0.021	0.119
Consumer knowledge	−0.223 **	0.136	−1.67	0.09	−0.076
Information	−0.560 ***	0.262	−2.13	0.033	−0.191
Source of information	0.490 ***	0.218	2.25	0.025	0.167
Quality	−0.266 **	0.148	−1.79	0.073	−0.091
Packaging	0.636 **	0.327	1.94	0.052	0.217
GM edible oil discount	0.223 **	0.117	1.85	0.058	0.076
Price	−0.372 ***	0.145	−2.55	0.011	−0.127
Advertisement	1.042 **	0.559	1.86	0.063	0.356
Place of supply and sale	−0.399 **	0.228	−1.74	0.081	−0.136
Oil-producing country	0.291 **	0.148	1.96	0.050	0.099

*** and ** indicates 5% and 10% significance level respectively.

**Table 5:** Goodness of fit measures for Probit model.

Log-Like Intercept only	−245.375
Log-Like Full Model	−208.579
LR (18)	73.591
LR (*p*-value)	0.000
McFadden’s $R^{2}$	0.150
ML (Cox–Snell) $R^{2}$	0.172
Cragg–Uhler $R^{2}$	0.240
Count $R^{2}$	0.726

**Table 6:** Estimation results of the second stage of the Tobit model.

Variables	Coefficients	Standard Deviation	T Stat.	Prob.
Age	0.008	0.005	1.51	0.133
Number of family members	0.092 **	0.049	1.85	0.065
Education	−0.098 ***	0.046	−2.11	0.036
Residential area	−0.054	0.040	−1.36	0.175
Monthly income	−0.198 **	0.118	−1.67	0.09
Monthly food expenses	0.912 **	0.535	1.70	0.089
Familiarity with GM products	−0.184	0.116	−1.59	0.114
Information	−0.352 ***	0.157	−2.23	0.026
Effect of oil brand	−0.278 **	0.165	−1.68	0.095
Price level	−0.35 ***	0.11	−3.04	0.003
Discounts	0.495 ***	0.181	2.73	0.007
Advertisement	0.265 ***	0.087	3.02	0.003
Trust in the government	0.145 **	0.086	1.69	0.093
Inverse Mills Ratio	0.366 **	0.20	1.86	0.08
Goodness of fit measures	R-Squared	Adjusted R-Squared	Std. Error or the Estimate	Durbin Watson
Stat.	0.286	0.183	1.0299	1.988

*** and ** indicates 5% and 10% significance level respectively.

## Data Availability

The data presented in this study are available on request from the corresponding author.
